# Severe Malaria Not Responsive to Artemisinin Derivatives in Man Returning from Angola to Vietnam

**DOI:** 10.3201/eid2107.150402

**Published:** 2015-07

**Authors:** Nguyen Van Hong, Alfred Amambua-Ngwa, Nguyen Quang Tuan, Do Duy Cuong, Nguyen Thi Huong Giang, Nguyen Van Dung, Ta Thi Tinh, Nguyen Van Tien, Bui Quang Phuc, Tran Thanh Duong, Anna Rosanas-Urgell, Jean-Pierre Van Geertruyden, Umberto D’Alessandro, Annette Erhart

**Affiliations:** National Institute of Malariology, Parasitology and Entomology, Hanoi, Vietnam (N. Van Hong, T.T. Tinh, B.Q. Phuc, T.T. Duong);; Medical Research Unit, Fajara, The Gambia (A. Amambua-Ngwa, U. D’Alessandro);; Bach Mai Hospital, Hanoi (N.Q. Tuan, D.D. Cuong, N.T.H. Giang, N. Van Dung, N. Van Tien);; Institute of Tropical Medicine, Antwerp, Belgium (A. Rosanas-Urgell, A. Erhart);; University of Antwerp (J.-P. Van Geertruyden)

**Keywords:** Plasmodium falciparum, malaria, artesunate, dihydroartemisinin, quinine, doxycycline, resistance, Africa, Angola, Vietnam, parasites

**In Response:** We agree with Ringwald and Dondorp (*1*) that our report of a Vietnamese worker returning from Angola with severe *Plasmodium falciparum* malaria not responsive to artemisinins (*2*) is unlikely to indicate that artemisinin resistance has reached Angola. Nevertheless, this case, for its unusual clinical manifestation and response to treatment, had raised alarm in Vietnam, where the number of imported malaria cases and deaths among Vietnamese workers returning from Africa has recently increased (*3**,**4*). After our report was published in July 2014, we collected additional information that may be useful in putting such a case in perspective. 

The results of an external quality control study by Sigma-Tau Pharmaceuticals on 10 vials of the same batch (no. 511002) of intravenous artesunate as administered to our case-patient (report available on request) confirmed acceptable drug concentration and showed that the opalescence observed after reconstitution was caused by precipitation of an impurity (representing 0.12% of the preparation) identified as an active metabolite of artesunate. Therefore, the treatment administered to the patient was of acceptable quality. The blood concentrations of artesunate and dihydroartemisinin may have been 20% lower than ideal (as predicted by a pharmacokinetic model), but this finding cannot explain why the parasite density remained >200,000/μL for several days.

Ringwald and Dondorp also mention functional asplenia as a possible cause of delayed parasite clearance. We argue that this would have resulted in a much longer (weeks/months) parasite clearance (*5*) than the observed sharp decrease after quinine and tetracycline administration. Moreover, we did not observe any accumulation of circulating dead parasites (Howell-Jolly bodies), which is against the hypothesis of functional asplenia. Furthermore, sharp decline of parasite density immediately after quinine and doxycycline administration by nasogastric tube is not consistent with the proposed hypothesis of reduced intestinal absorption.

In hindsight and after consideration of additional information, we agree that it is unlikely this patient harbored several resistant parasite clones. However, the reasons for the lack of response to arteminins in this patient remain unknown and are under continued investigation.

The discussion triggered by the publication of our case report raises the question of what should be reported to the attention of the scientific community and public health authorities. Besides being an obligation for clinical physicians, reporting unusual treatment failures such as our case is also an essential component of antimalarial resistance surveillance. As mentioned by Ringwald and Dondorp, “vigilant monitoring is pivotal” for the detection of possible foci of resistance. For early detection of artemisinin resistance, we would rather have a more sensitive than specific system, because the latter would probably miss the first emerging cases of resistance. Reporting cases similar to the one we published should be encouraged.
